# *Staphylococcus aureus* Alpha-Toxin Limits Type 1 While Fostering Type 3 Immune Responses

**DOI:** 10.3389/fimmu.2020.01579

**Published:** 2020-08-07

**Authors:** Agnes Bonifacius, Oliver Goldmann, Stefan Floess, Silva Holtfreter, Philippe A. Robert, Maria Nordengrün, Friederike Kruse, Matthias Lochner, Christine S. Falk, Ingo Schmitz, Barbara M. Bröker, Eva Medina, Jochen Huehn

**Affiliations:** ^1^Department Experimental Immunology, Helmholtz Centre for Infection Research, Braunschweig, Germany; ^2^Department Infection Immunology, Helmholtz Centre for Infection Research, Braunschweig, Germany; ^3^Department of Immunology, University Medicine Greifswald, Greifswald, Germany; ^4^Department Systems Immunology and Braunschweig Integrated Centre of Systems Biology, Helmholtz Centre for Infection Research, Braunschweig, Germany; ^5^Institute of Infection Immunology, TWINCORE, Centre for Experimental and Clinical Infection Research; A Joint Venture Between the Medical School Hannover and the Helmholtz Centre for Infection Research, Hanover, Germany; ^6^Institute of Medical Microbiology and Hospital Epidemiology, Hannover Medical School, Hanover, Germany; ^7^Institute of Transplant Immunology, Hannover Medical School, Hanover, Germany; ^8^DZIF, German Center for Infectious Diseases, TTU-IICH Hannover-Braunschweig Site, Hanover, Germany; ^9^Department Systems-Oriented Immunology and Inflammation Research, Helmholtz Centre for Infection Research, Braunschweig, Germany; ^10^Institute for Molecular and Clinical Immunology, Medical Faculty, Otto-von-Guericke-University Magdeburg, Magdeburg, Germany; ^11^Department of Molecular Immunology, Ruhr-University Bochum, Bochum, Germany

**Keywords:** *Staphylococcus aureus*, CD4^+^ T cells, alpha-toxin, innate lymphoid cells, γδ T cells

## Abstract

*Staphylococcus aureus* can cause life-threatening diseases, and hospital- as well as community-associated antibiotic-resistant strains are an emerging global public health problem. Therefore, prophylactic vaccines or immune-based therapies are considered as alternative treatment opportunities. To develop such novel treatment approaches, a better understanding of the bacterial virulence and immune evasion mechanisms and their potential effects on immune-based therapies is essential. One important staphylococcal virulence factor is alpha-toxin, which is able to disrupt the epithelial barrier in order to establish infection. In addition, alpha-toxin has been reported to modulate other cell types including immune cells. Since CD4^+^ T cell-mediated immunity is required for protection against *S. aureus* infection, we were interested in the ability of alpha-toxin to directly modulate CD4^+^ T cells. To address this, murine naïve CD4^+^ T cells were differentiated *in vitro* into effector T cell subsets in the presence of alpha-toxin. Interestingly, alpha-toxin induced death of Th1-polarized cells, while cells polarized under Th17 conditions showed a high resistance toward increasing concentrations of this toxin. These effects could neither be explained by differential expression of the cellular alpha-toxin receptor ADAM10 nor by differential activation of caspases, but might result from an increased susceptibility of Th1 cells toward Ca^2+^-mediated activation-induced cell death. In accordance with the *in vitro* findings, an alpha-toxin-dependent decrease of Th1 and concomitant increase of Th17 cells was observed *in vivo* during *S. aureus* bacteremia. Interestingly, corresponding subsets of innate lymphoid cells and γδ T cells were similarly affected, suggesting a more general effect of alpha-toxin on the modulation of type 1 and type 3 immune responses. In conclusion, we have identified a novel alpha-toxin-dependent immunomodulatory strategy of *S. aureus*, which can directly act on CD4^+^ T cells and might be exploited for the development of novel immune-based therapeutic approaches to treat infections with antibiotic-resistant *S. aureus* strains.

## Introduction

The gram-positive bacterium *Staphylococcus aureus* is an extracellular pathogen with the ability to invade and persist within host cells. *S. aureus* is known to cause severe infections, ranging from wound infections, endocarditis, and pneumonia to sepsis, and still represents a global public health threat due to its resistance toward various antibiotics (1–5). Although many attempts have been made, there is currently no vaccine available that can prevent *S. aureus* infections in humans. This might be due to efficient bacterial virulence and immune evasion mechanisms that enable *S. aureus* to escape immune surveillance by the host (6). Unraveling these mechanisms will be crucial for the development of novel immune-based adjunctive therapies and more efficient vaccines.

Numerous studies have reported a role of CD4^+^ T cells in anti-staphylococcal immunity. While Choi and colleagues have shown in a mouse model that vaccination with extracellular vesicles derived from *S. aureus* mediates protection against lethal lung infection through the action of IFNγ-producing CD4^+^ T helper 1 (Th1) cells (7), others have reported a role for both Th1 cells and IL-17A-producing CD4^+^ T helper 17 (Th17) cells in vaccine-mediated protection against *S. aureus* bloodstream infection (8). Yet, another study suggested that immunization with a multicomponent vaccine protected mice in a kidney abscess model as well as a peritonitis model through the synergistic action of Th17 cells and antibodies (9). These and other examples clearly show that, depending on the vaccination approach and the utilized infection model, different CD4^+^ effector T cell subsets can confer protection against *S. aureus* (10, 11).

*S. aureus* is producing a variety of extracellular virulence factors, and for some of these, immunomodulatory properties have already been described. Toxic shock syndrome toxin 1, which is one example for a staphylococcal superantigen, is causing polyclonal T cell activation, resulting in overwhelming inflammation (12). Other secreted proteins result in allergic responses or favor regulatory T cell differentiation (13–15). Very recently, Richardson et al. have shown that staphylococcal phenol-soluble-modulins inhibit *in vivo* Th1 and Th17 polarization during systemic *S. aureus* infection (16). One major virulence factor of *S. aureus* is alpha-toxin (aka alpha-hemolysin, hla), which was first described for its lytic activity toward rabbit erythrocytes (17). Alpha-toxin is secreted as a monomer and forms heptameric pores upon binding to the host cell membrane, resulting in death of the target cell (18). The initial binding step is mediated by the cellular factor ADAM10, a disintegrin and metalloproteinase domain-containing protein 10 (19). At sublytic concentrations, alpha-toxin was shown to impact signaling pathways in different cell types, hinting toward modulatory properties besides its cytolytic activity (19–21).

While previous studies have reported an induction of IFNγ and IL-17A production by human CD4^+^ T cells stimulated with sublytic concentrations of alpha-toxin (22–24), the direct immunomodulatory activity of alpha-toxin on CD4^+^ T cell differentiation was not studied, yet. Thus, we here cultured naïve CD4^+^ T cells under polarizing conditions and analyzed the impact of alpha-toxin on survival, proliferation and differentiation of the cells. Unexpectedly, we found differential survival of Th1 and Th17 cells cultured in presence of alpha-toxin. While death of Th1 cells was dose-dependent, Th17 cells were more resistant toward alpha-toxin. Mechanistically, we could rule out an involvement of ADAM10 and caspases. When we translated these *in vitro* findings into an *in vivo* setting, we observed an alpha-toxin-dependent decrease of Th1 cells and a concomitant increase of Th17 cells during *S. aureus* bacteremia. The finding that corresponding subsets of innate lymphoid cells (ILCs) and γδ T cells were similarly affected, suggested that *S. aureus* has evolved a general strategy to modulate type 1 and type 3 immune responses in addition to its specific targeting of Th1 cells.

## Materials and Methods

### Animals

B6.Foxp3^tm1(CD2/CD52)Shori^ mice, Foxp3^hCD2^ reporter mice on C57BL/6 background expressing human CD2 (hCD2) under control of the *Foxp3* locus (25), and wildtype C57BL/6J mice were bred and housed under specific pathogen-free conditions at the animal facility of the Helmholtz Center for Infection Research (Braunschweig, Germany). Animals were handled in accordance with good animal practice as defined by FELASA and the national animal welfare body GV-SOLAS guidelines. All animal experiments were approved by the Lower Saxony Committee on the Ethics of Animal Experiments as well as the responsible state office (Lower Saxony State Office of Consumer Protection and Food Safety) under the permit number 33.19-42502-04-13/1195. For all *in vivo* experiments, gender- and age-matched animals were used.

### Recombinant *S. aureus* Proteins

Recombinant alpha-toxin (Pan-genome identifier: SAUPAN003418000; annotation in *S. aureus* USA300: SAUSA300_1058), Lipase (Lip, aka Sal1, pan-genome identifier: SAUPAN006420000, annotation in *S. aureus* USA300: SAUSA300_2603 - lip) and Phospholipase C (Plc; pan genome identifier: SAUPAN000858000; annotation in *S. aureus* USA300: SAUSA300_99 - plc) (26) were produced and purified as described before (24). In brief, corresponding gene sequences of *S. aureus* strain USA300 were introduced into an *Escherichia coli* (*E. coli*) plasmid (pPR-IBA1) with strep-tag II (IBA). Following plasmid amplification in *E. coli* DH5α, the proteins were overexpressed in *E. coli* BL21 pLysS. Utilizing strep-Tactin Superflow columns (IBA) and EndoTrap Red Columns (Profox), recombinant *S. aureus* proteins were purified and LPS contamination minimized.

### Antibodies

The following antibody conjugates (all purchased from BioLegend, eBioscience, or BD Biosciences) were used for flow cytometry and cell sorting: anti-hCD2-APC (RPA-2.10), anti-CD3-BV510 (17A2), anti-CD3-APC (17A2), anti-CD4-BV605 (RM4-5), anti-CD4-PE/Cy7 (RM4-5), anti-CD8α-APC (53-6.7), anti-CD11c-APC (N418), anti-CD19-APC (6D5), anti-CD45R-APC (RA3-6B2), anti-CD62L-BV605 (MEL-14), anti-CD127-PE (A7R34), anti-F4/80-APC (BM8), anti-Gr1-APC (RB6-8C5), anti-γδTCR-FITC (eBioGL3), anti-Ter119-APC (TER-119), anti-RORγt-PE (AFKJS-9), anti-RORγt-BV421 (Q31-378), anti-T-bet-PE/Cy7 (4B10), anti-T-bet-PE (4B10), anti-Gata3-PerCP/eFluor710 (TWAJ), anti-IFNγ-FITC (XMG1.2), anti-IFNγ-BV785 (XMG1.2), anti-IL-17-PE (eBio17B7), and anti-IL-17A-APC (TC11-18H10.1). Anti-ADAM10-PE (139712) was purchased from R&D. Exact staining panels for the different experimental settings are presented in Table S1. All antibodies were titrated prior to use to determine optimal concentration for staining.

### Flow Cytometry

Flow cytometry analysis was performed as recently described (27). Prior to intracellular cytokine staining, cells were incubated with phorbol 12-myristate 13-acetate (PMA, 10 ng/ml), Ionomycin (0.5 μg/ml), and Brefeldin A (10 μg/ml) for 4 h at 37°C (all Sigma-Aldrich). For detection of cell survival and dead cell exclusion, cells were incubated with a UV-excitable, fixable Live/Dead dye (LIVE/DEAD™ Fixable Blue Dead Cell Stain Kit, Life Technologies) for 30 min. Surface staining was performed for 15 min on ice in PBS (Gibco) containing 0.2 % bovine serum albumin (BSA, Sigma-Aldrich). Subsequently, cells were fixed, permeabilized using the Foxp3 Transcription Factor Staining Kit (eBioscience) according to the manufacturer's protocol, and stained intracellularly for cytokines and transcription factors for 30 min or overnight. For detection of caspase-3/7 activity, cells were harvested, washed twice with PBS and dead cells were labeled with a UV-excitable, fixable Live/Dead dye for 30 min. The staining reaction was stopped by washing with PBS/BSA. Subsequently, 50 μl of CellEvent Caspase-3/7 Green Detection Reagent (Thermo Fisher) was added to each sample. After 30 min of incubation at 37°C, cells were kept on ice until acquisition. All flow cytometry samples were acquired at BD FACS LSR-II SORP or BD LSR-Fortessa (BD Biosciences), and data were analyzed with FlowJo® 9.9.6 (FlowJo, LLC).

### *In vitro* CD4^+^ T Cell Differentiation

To sort highly pure naïve CD4^+^ T cells devoid of any Foxp3^+^ regulatory T cells, we made use of Foxp3^hCD2^ reporter mice (25). Single cell suspensions were prepared from spleen and lymph nodes (LN) by using a 100 μm cell strainer, and cleared from erythrocytes by incubation with ammonium chloride buffer at room temperature for 3 min. In most experiments, CD4^+^ T cells were first magnetically enriched by anti-CD4 microbeads using the autoMACS® Pro (Miltenyi Biotec). Subsequently, naïve CD4^+^ T cells were sorted by fluorescence-activated cell sorting (FACS) as Foxp3^hCD2−^CD62L^high^CD4^+^Lin^−^ cells (Lin: CD8α, CD45R, CD11c, F4/80) utilizing a BD FACSAria-Ilu (BD Biosciences), BD FACS Aria-II SORP (BD Biosciences) or MoFlo XDP (Beckman Coulter). In the experiments assessing ADAM10 expression, conventional Foxp3^hCD2−^CD4^**+**^ T cells were isolated by magnetic-activated cell sorting (MACS). In brief, cells were first stained with anti-hCD2-APC and subsequently magnetically separated by anti-APC Microbeads (Miltenyi Biotec) using the autoMACS® Pro. Next, CD4^+^ T cells were sorted from the hCD2^−^ fraction by anti-CD4 microbeads using the autoMACS® Pro. In some experiments, sorted naïve CD4^+^ T cells were stained with the proliferation dye CellTrace™ Violet (CTV, Thermo Fisher Scientific) prior to cultivation. Naïve CD4^+^ T cells were cultured in IMDM containing 10 % FCS, 1 mM sodium pyruvate, 50 U/ml Penicillin and Streptomycin, 25 mM HEPES, 50 μM beta-mercaptoethanol, and non-essential amino acids (Gibco Life Technologies and Biochrom AG) (cIMDM), and 150,000 cells per well were seeded into 96-well flat bottom plates (Thermo Fisher Scientific) previously coated with 1 μg/ml anti-CD3 (17A2, BioLegend) and 1 μg/ml anti-CD28 (37.51, BioLegend). For polarization into different CD4^+^ effector T cell subsets, the following cytokines and antibodies were added: Th1 – IL-12 (20 ng/ml), anti-IL-4 (10 ng/ml, 11B11); Th17 – IL-6 (30 ng/ml), TGFβ1 (2 ng/ml), IL-1β (10 ng/ml), anti-IL-2 (5 ng/ml, JES6-1A12), anti-IFNγ (10 ng/ml, XMG1.2) (all purchased from BioLegend, BioXCell, Peprotech or R&D). Furthermore, recombinant *S. aureus* proteins or Ionomycin (Sigma-Aldrich) were added to the cultures at indicated concentrations. GI254023X (10 nM, Sigma Aldrich) was added to the cultures at day 0, while Q-VD-OPh (Q-VD, 20 μM, MP Biomedicals) was supplied daily. Cells were incubated for indicated time points. If analyzed later than day 3, cells were removed from the stimulus on day 3 and transferred to an uncoated plate. At this step, Th1 cells received IL-2 (5 ng/ml, R&D Systems), and Th17 cells received IL-23 (10 ng/ml, BioLegend). To analyze the impact of alpha-toxin on already differentiated effector T cells, Th1 and Th17 cells were generated as described above. On day 5, cells were harvested, counted and either kept in culture (96-well plates) without restimulation or restimulated in 96-well plates coated with 0.1 μg/ml anti-CD3, 1 μg/ml anti-CD28. Part of the cells received 1,000 ng/ml alpha-toxin, and Th1 and Th17 cells were supplemented with IL-2 and IL-23, respectively. After 24 or 48 h, cell survival was analyzed via flow cytometry as described before.

### Systemic *S. aureus* Infection

Systemic infection with *S. aureus* was performed as previously described (28). In brief, 40 ml of Brain Heart Infusion medium (BHI; Roth) were inoculated with few colonies of wildtype *S. aureus* 6850 (hereafter called wildtype) or an isogenic Δhla mutant (hereafter called Δhla). Following overnight incubation at 120 rpm at 37°C, bacteria were diluted 1:10 in BHI medium and further cultivated. Every hour, the optical density (OD) at 600 nm was measured until an OD of 0.5 was reached. Bacteria were washed twice with PBS to remove toxins, resuspended in PBS and the OD was adjusted to 1. Aliquots of 0.5 ml were prepared and stored at −80°C until use. Following infection, serial dilutions were prepared from the remaining bacterial stock and plated on COL blood agar plates. The plates were incubated overnight at 37°C and colony-forming units (CFU) counted the next day. Female C57BL/6J mice (age 8–16 weeks) were infected i.v. with 3.5 × 10^6^ CFU of wildtype or ΔHla *S. aureus*. Body weight and general health status as well as the occurrence of superficial lesions were monitored daily for 2 weeks. Animals were sacrificed and excluded from analysis if weight loss exceeded 20 %. On day 14 post infection (p.i.), mice were sacrificed, inguinal LNs and spleens were collected and meshed through 100 μm cell strainers to obtain single cell suspensions for flow cytometric analysis. Splenocytes were cleared from erythrocytes by incubation with ammonium chloride buffer at room temperature for 3 min. Cell numbers were counted at Accuri C6 cytometer (BD Biosciences). Furthermore, blood was collected and incubated at room temperature for a minimum of 15 min, followed by centrifugation at 2,000 g for 10 min. Serum was collected and stored at −20°C until further processing.

### Cytokine/Chemokine Measurement

Serum samples (30 μl diluted 1+1 with sample diluent) were analyzed with Bio-Plex Pro Mouse Cytokine 23-plex Assay Kit (Bio-Rad Laboratories) according to the manufacturer's instructions.

### Statistical Analysis

Graphs were generated and statistical analyses were performed with Prism 5.0 and 8.2 (GraphPad Software). Comparisons of more than two groups were carried out by Kruskal-Wallis test, followed by Dunn's multiple comparison test. For time- or concentration-dependent comparison of two groups, Two-way ANOVA, followed by Bonferroni multiple comparisons, was applied. *P*-values below 0.05 were considered significant with ^*^p < 0.05, ^**^p < 0.01, ^***^p < 0.001, ^****^p < 0.0001.

### Institutional Safety Regulation and Biosecurity

All work on genetically modified cells and organisms was carried out in properly regulated laboratories according to the regulations stipulated by the health and safety executive of each institution. Pathogens were handled in laboratories and animal housing facilities registered according to the German Act on Genetic Engineering and categorized as safety level 2. Permissions for constructing and handling genetically engineered cells and organisms were obtained from the relevant national authorities. Projects, project leaders, and safety executives were registered to the authorities. Unauthorized distribution of *S. aureus* or *S. aureus* recombinant proteins was restricted by the following measures: 24 h surveillance of the institutional premises; electronic access control at the gate, at each building, and within each building; accordingly labeled freezers for storage; access for trained staff only; distribution of pathogens within Germany only to license holders according to §44 Infection Protection Act and according to the European Agreement concerning the International Carriage of Dangerous Goods by Road (ADR) and the IATA Dangerous Goods Regulation (DGR).

## Results

### Alpha-Toxin Induces Death of Th1 Cells, While Th17 Are More Resistant

**Figure 1:**
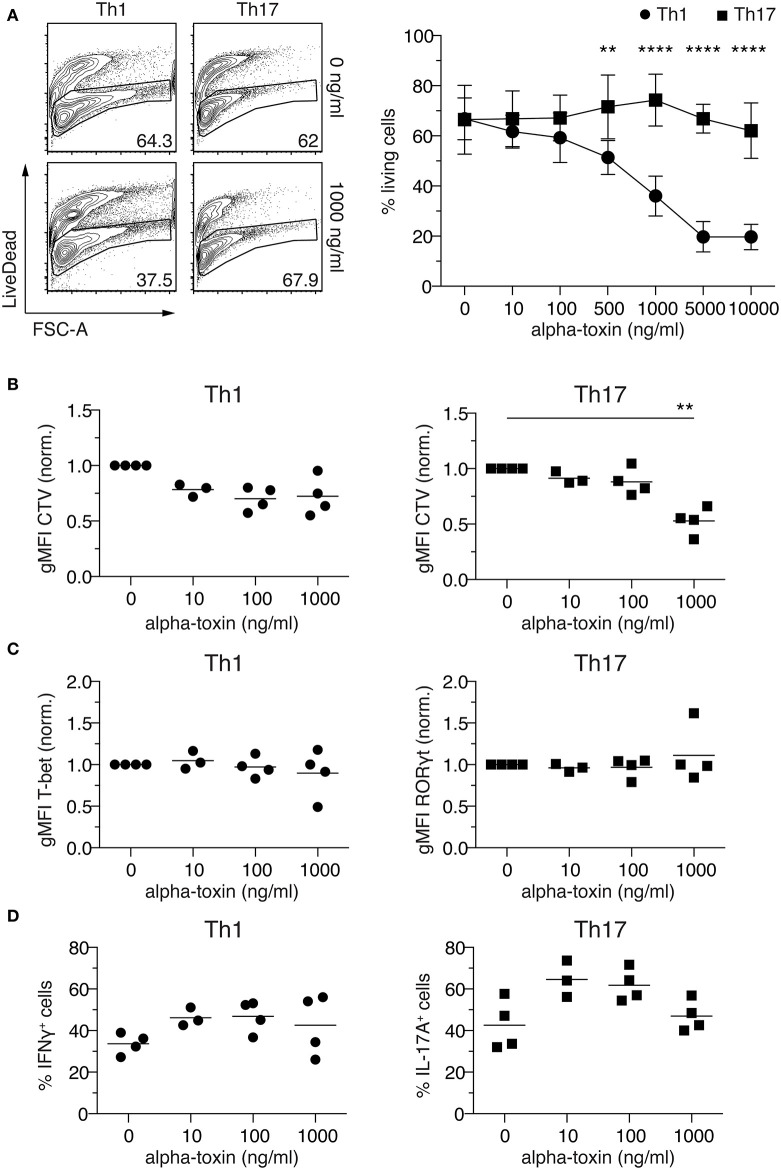
Alpha-toxin induces differential survival in Th1 and Th17 cells. Murine naïve CD4^+^ T cells were differentiated *in vitro* into Th1 or Th17 cells in presence of increasing concentrations of alpha-toxin. On day 4 of the culture, cells were analyzed by flow cytometry. **(A)** Left: Representative contour plots show frequencies of living cells in indicated cultures. Numbers indicate frequencies of cells in gates. Right: Graph summarizes frequencies of living cells among Th1- (circles) and Th17-polarized (squares) cells (mean ± SD). **(B)** gMFI of CellTrace™ Violet indicates proliferation of Th1 (left) and Th17 cells (right) in presence of indicated concentrations of alpha-toxin. Cells were gated on living CD4^+^ T cells and data were normalized to the medium control. **(C)** gMFIs of T-bet expression in Th1 cells (left) and RORγt expression in Th17 cells (right) are depicted. Cells were gated on living CD4^+^ T cells and data were normalized to the medium control. **(D)** Frequencies of IFNγ^+^ cells among Th1 cells (left) and IL-17A^+^ cells among Th17 cells (right) are depicted. Cells were gated on living CD4^+^ T cells. **(A–D)** Data were pooled from 3–5 independent experiments with technical triplicates. For statistical analysis, Two-way ANOVA, followed by Bonferroni multiple comparisons **(A)** or Kruskal-Wallis test, followed by Dunn's multiple comparison test **(B)** was applied. gMFI, Geometric mean fluorescence intensity. **p < 0.01, ****p < 0.0001.

Sublytic concentrations of alpha-toxin were previously reported to induce IFNγ and IL-17A production by human CD4^+^ T cells (22–24), yet the direct immunomodulatory activity of alpha-toxin on CD4^+^ T cell differentiation was not studied, so far. To unravel whether staphylococcal alpha-toxin can directly modulate CD4^+^ T cell differentiation, naïve Foxp3^−^CD62L^high^CD4^**+**^ T cells were isolated from Foxp3^hCD2^ reporter mice and cultured *in vitro* under Th1- and Th17-polarizing conditions in antigen-presenting cell-free cultures. The selected culture conditions resulted in a homogenous expression of T-bet in Th1-polarized cells and, particularly during the first days of differentiation, a homogenous expression of RORγt in Th17-polarized cells (Figures S1A–D). At day 4, a still early time point of T cell differentiation, substantial fractions of the cells cultured under Th1- and Th17-polarizing conditions already showed IFNγ and IL-17A expression, respectively (Figures S1B,C), in line with recent studies involving *in vitro* T cell differentiation cultures (29, 30). From here on, cells cultured under Th1- and Th17-polarizing conditions will be referred to as Th1 and Th17 cells, respectively. To assess the impact of alpha-toxin on CD4^+^ T cell differentiation, cells were cultured under Th1- and Th17-polarizing conditions in presence of increasing concentrations of alpha-toxin, and T cell survival as well as differentiation was assessed by flow cytometry on day 4 of the cultures. Interestingly, we observed a differential impact of alpha-toxin on the survival of Th1 and Th17 cells. When cultured under Th1-polarizing conditions, increasing concentrations of alpha-toxin resulted in a dose-dependent decrease in the frequency of viable cells, while cells cultured under Th17-polarizing conditions were not affected in their viability even at high alpha-toxin concentrations (Figure 1A). Yet, alpha-toxin induced an enhanced proliferation of Th17 cells as indicated by significantly reduced geometric mean fluorescence intensity (gMFI) of the proliferation dye CTV. However, proliferation of cells cultured under Th1-polarizing conditions was only slightly affected, without reaching statistical significance (Figure 1B and Figures S1B,C). Next, we studied the impact of alpha-toxin on the differentiation of Th1 and Th17 cells by measuring the expression levels of the lineage specification factors T-bet and RORγt, respectively. When gating on the viable cells at day 4 of the culture, hardly any effect of alpha-toxin on the expression of the key transcription factors could be observed (Figure 1C and Figures S1B,C). In contrast, mild effects could be observed with regard to the expression of effector cytokines by the two T cell subsets. Here, alpha-toxin resulted in slightly increased frequencies of IFNγ^+^ Th1 cells and IL-17A^+^ Th17 cells, without reaching statistical significance (Figure 1D and Figures S1B,C). To rule out any unspecific effects of the recombinantly produced alpha-toxin, control experiments with similarly produced recombinant *S. aureus* proteins were performed. Neither Lip nor Plc had any impact on Th1 or Th17 cell differentiation with regards to viability (Figure S2A) and cytokine production (Figure S2B). In summary, CD4^+^ T cells cultured under Th1- and Th17-polarizing conditions showed differential susceptibility toward alpha-toxin, with Th1 cells being highly susceptible and Th17 cells more resistant to the toxin-induced cell death.

### Increased Cell Death of Th1 Cells in Response to Alpha-Toxin Does Not Result From Higher ADAM10 Expression Levels

**Figure 2:**
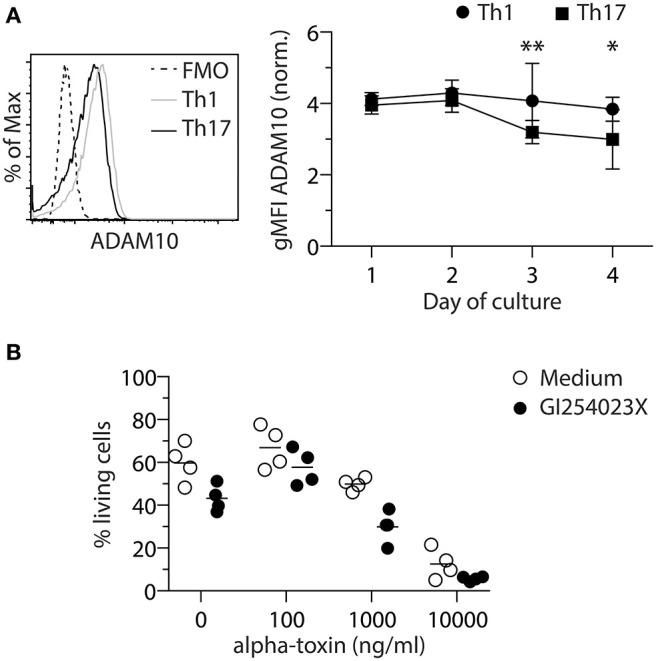
ADAM10 does not play a role in alpha-toxin-mediated death of Th1 cells. Murine conventional CD4^+^ T cells were differentiated into Th1 or Th17 cells and analyzed for ADAM10 expression by flow cytometry. Representative histogram and the summarizing graph show expression level of ADAM10 relative to FMO control. Cells were gated on living cells **(A)**. Murine naïve CD4^+^ T cells were differentiated into Th1 cells in presence of indicated concentrations of alpha-toxin and in presence or absence of the ADAM10 inhibitor GI254023X. On day 4 of the culture, survival of the cells was analyzed by flow cytometry. Summarizing graph shows frequencies of living cells under the different conditions **(B)**. Data were pooled from 4–9 independent experiments with 2–3 technical replicates. For statistical analysis, Two-way ANOVA, followed by Bonferroni multiple comparisons was applied. gMFI - Geometric mean fluorescence intensity. *p < 0.05, **p < 0.01.

Secreted monomeric alpha-toxin can bind to ADAM10 on target cells and subsequently form heptameric pores, leading to cell death (19). To investigate if the observed differential susceptibility of Th1 and Th17 cells toward alpha-toxin is associated with a differential expression of ADAM10, Foxp3^hCD2−^ conventional CD4^+^ T cells were differentiated *in vitro* into Th1 or Th17 cells and ADAM10 expression was analyzed daily for 4 days by flow cytometry. On days 3 and 4, Th1 cells displayed slightly higher levels of ADAM10 as compared to Th17 cells (Figure 2A). To investigate whether this differential ADAM10 expression would affect alpha-toxin-mediated cell death, we made use of an ADAM10 inhibitor, GI254023X, which has been reported to prevent binding of alpha-toxin to ADAM10 in a number of studies (31–34). However, inhibition of ADAM10 by GI254023X did not restore survival of Th1 cells (Figure 2B). Taken together, we could show that although Th1-polarized cells express slightly higher levels of ADAM10 when compared to Th17-polarized cells, the interaction between alpha-toxin and ADAM10 is not causing the differential survival of Th1 and Th17 cells cultured in presence of alpha-toxin.

### Caspase Inhibition Does Not Prevent Death of Th1 Cells in Response to Alpha-Toxin

**Figure 3:**
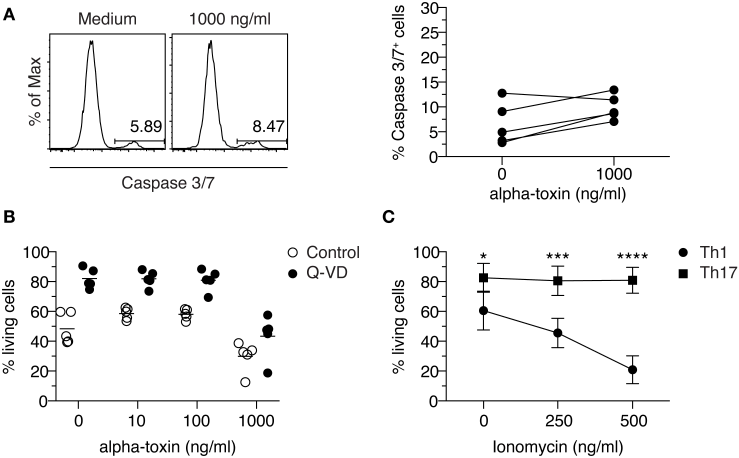
Th1 cells cannot be rescued from alpha-toxin-mediated cell death by Caspase inhibition. Murine naive CD4^+^ T cells were differentiated into Th1 cells in presence or absence of alpha-toxin and Caspase 3/7 activity was assessed by flow cytometry on day 3 of the culture. Representative histograms and the summarizing graph show frequencies of cells with active Caspase 3/7, gated on living cells **(A)**. Murine naïve CD4^+^ T cells were differentiated into Th1 cells in presence of indicated concentrations of alpha-toxin and in presence or absence of the pan-caspase inhibitor Q-VD. On day 4 of the culture, survival of the cells was analyzed by flow cytometry. Summarizing graph shows frequencies of living cells under the different conditions **(B)**. Murine naïve CD4^+^ T cells were differentiated into Th1 or Th17 cells in presence of indicated concentrations of Ionomycin. On day 3 of the culture, survival of the cells was analyzed by flow cytometry. Summarizing graph shows frequencies of living cells under the different conditions **(C)**. Data are pooled from 3–5 independent experiments with technical triplicates. For statistical analysis, Two-way ANOVA, followed by Bonferroni multiple comparisons was applied. *p < 0.05, ***p < 0.001, ****p < 0.0001.

It has been reported that human T cells die from programmed cell death upon short-term exposure to alpha-toxin (35). To investigate if Th1-polarized cells cultured in presence of alpha-toxin die from apoptosis, we analyzed the activity of caspase-3/7 in those cells. To this end, naïve CD4^+^ T cells were differentiated into Th1 cells in presence of different concentrations of alpha-toxin. On day 3 of the culture, 1 day before differential survival of Th1 and Th17 cells cultured in presence of alpha-toxin was observed, the frequency of caspase-3/7^+^ (apoptotic) cells was assessed by flow cytometry. The frequencies of apoptotic cells were very low in cells cultured in medium control and did not increase significantly in presence of 1,000 ng/ml alpha-toxin (Figure 3A). Since caspase activation was only analyzed at one time point, we were interested to see whether an inhibition of caspases over the complete time could rescue the Th1-polarized cells from alpha-toxin-mediated cell death. In accordance with the low frequencies of apoptotic cells on day 3, the cell survival could not be rescued by addition of the pan-Caspase inhibitor Q-VD to the culture (Figure 3B). We could show that Th1-polarized cells cultured in presence of alpha-toxin express only low levels of caspases and can furthermore not be rescued from alpha-toxin-induced cell death via inhibition of caspases.

### Th1 Cells Are More Susceptible to Ionomycin-Mediated Activation-Induced Cell Death When Compared to Th17 Cells

Alpha-toxin can induce an increase of intracellular Ca^2+^ levels (36). To study if Th1 cells show an increased susceptibility toward elevated intracellular Ca^2+^ levels when compared to Th17 cells, naïve CD4^+^ T cells were differentiated into Th1 or Th17 cells in presence of the Ca^2+^ ionophore Ionomycin. On day 3 of the cultures, the frequency of viable cells was determined by flow cytometry. While the addition of 500 ng/ml Ionomycin to the cultures resulted in a decreased frequency of viable cells cultured under Th1-polarizing conditions, cells cultured under Th17-polarizing conditions remained unaffected (Figure 3C), suggesting an increased susceptibility of Th1 cells toward Ca^2+^-mediated activation-induced cell death. Interestingly, addition of alpha-toxin during the restimulation of already differentiated Th17 cells did not result in a decreased frequency of viable cells, supporting the finding that Th17 cells are resistant to the toxin-induced cell death. Yet, it could not be confirmed that also already differentiated Th1 cells display a higher susceptibility toward alpha-toxin as the rapid and strong activation-induced cell death that was induced under the applied restimulation conditions could not be further increased upon addition of alpha-toxin (Figure S3).

### Alpha-Toxin Causes a Reduced Th1 and an Increased Th17 Response Upon *S. aureus* Infection

**Figure 4:**
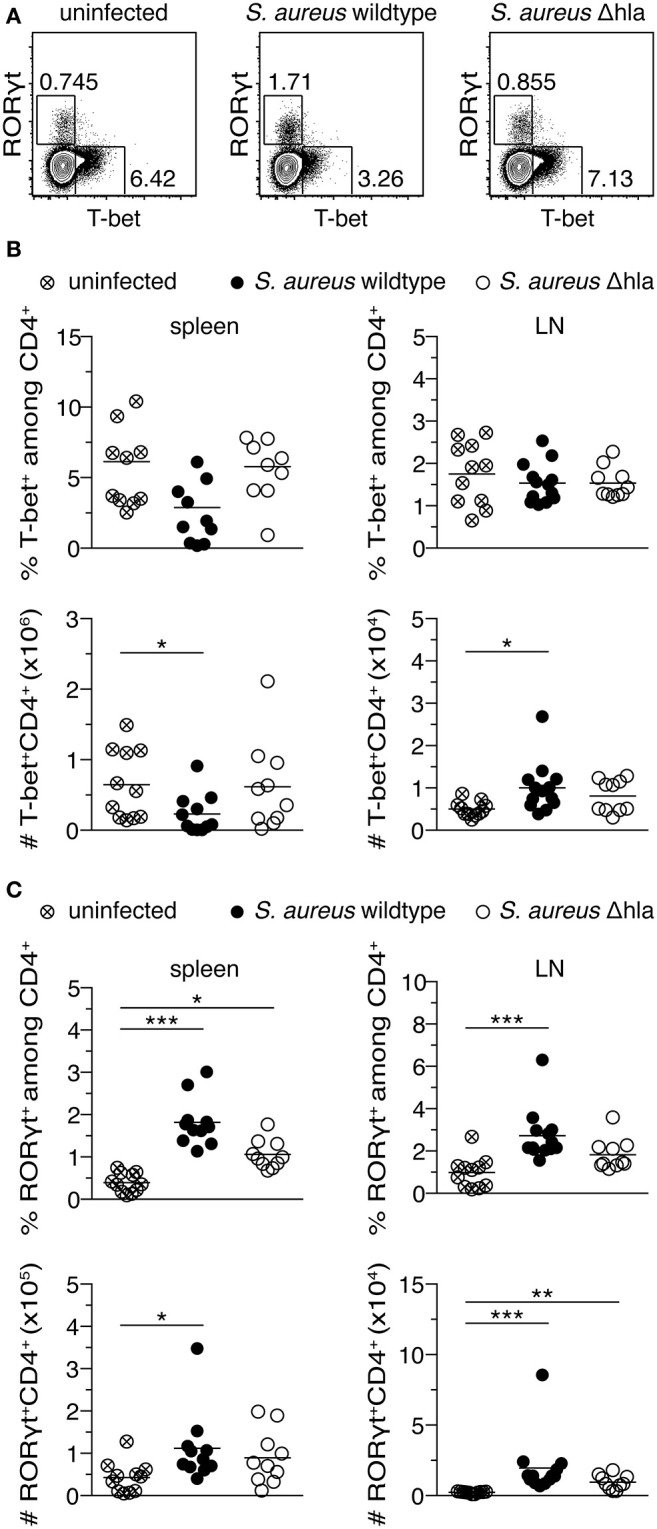
*S. aureus* alpha-toxin shifts CD4^+^ helper T cells toward Th17 cells. Female C57BL/6J mice were either left uninfected or infected i.v. with *S. aureus* wildtype or Δhla. On day 14 p.i., animals were sacrificed and organs harvested for flow cytometric analysis. Representative contour plots show T-bet and RORγt expression among splenic CD4^+^ T cells **(A)**. Frequencies of T-bet^+^ cells among CD4^+^ T cells in spleen and LNs and their absolute numbers are depicted **(B)**. Frequencies of RORγt^+^ cells among CD4^+^ T cells in spleen and LNs and their absolute numbers are depicted **(C)**. Data were pooled from two independent experiments and each dot represents one animal. For statistical analysis, Kruskal-Wallis test, followed by Dunn's multiple comparison test, was applied. *p < 0.05, **p < 0.01, ***p < 0.001.

We have shown that Th1-polarized cells are more susceptible toward alpha-toxin *in vitro* when compared to Th17-polarized cells. To demonstrate a role of the different susceptibilities of T cell subtypes toward alpha-toxin *in vivo*, female C57BL/6J mice were infected i.v. with a *S. aureus* wildtype strain or an isogenic Δhla mutant strain. The animals were monitored daily for a time period of 14 days. Although both groups displayed similar survival and body weight changes (Figure S4A), animals infected with *S. aureus* wildtype had an increased risk to develop superficial tissue lesions (abscesses) close to their tail base during the course of infection as compared to animals infected with *S. aureus* Δhla (Figure S4A). As expected, sera of infected animals showed increased levels of various inflammatory cytokines and chemokines compared to uninfected controls (Figure S5). Interestingly, no overt differences between animals infected with *S. aureus* wildtype and *S. aureus* Δhla were detected. However, we observed substantial changes within the CD4^+^ T cell compartment 14 days p.i., a time point at which the adaptive immune response becomes clearly visible. Upon infection with *S. aureus* wildtype, RORγt^+^ (Th17) cells inside spleen and LNs were significantly increased both in frequencies and numbers, while the absolute number of T-bet^+^ (Th1) cells was significantly decreased in the spleen (Figure 4 and Figure S4B). In animals infected with *S. aureus* Δhla, Th1 frequencies and absolute numbers were similar to uninfected controls in both organs (Figure 4B). The frequencies and numbers of Th17 cells inside spleen and LNs were slightly lower in these animals as compared to *S. aureus* wildtype-infected animals, albeit not as low as in uninfected controls (Figure 4C). Of note, total cellularity of the spleen was similar in all experimental groups, while cell numbers within LNs were significantly enhanced upon infection with *S. aureus* wildtype and to a lesser extent with *S. aureus* Δhla when compared to uninfected controls (Figure S4D). On the level of effector cytokine expression, for the Th1 lineage no differences in the frequencies of IFNγ^+^ cells could be observed in spleen and LNs (Figures S6A,B), despite the abovementioned decrease in the number of T-bet^+^ cells, suggesting a T-bet-independent IFNγ expression as reported before (37). For the Th17 lineage, the frequencies of IL-17A^+^ cells are reflecting the changes of RORγt^+^ cells (Figures S6A,C). To conclude, we have found that alpha-toxin not only has a direct impact on CD4^+^ T cells *in vitro* but can also modulate the CD4^+^ T cell response *in vivo* by promoting Th17 cells and limiting Th1 cells.

### Alpha-Toxin Reduces Frequencies of Both T-bet^+^ γδ T Cells and Type 1 ILCs, While Increasing Frequencies of RORγt^+^ γδ T Cells *in vivo*

**Figure 5:**
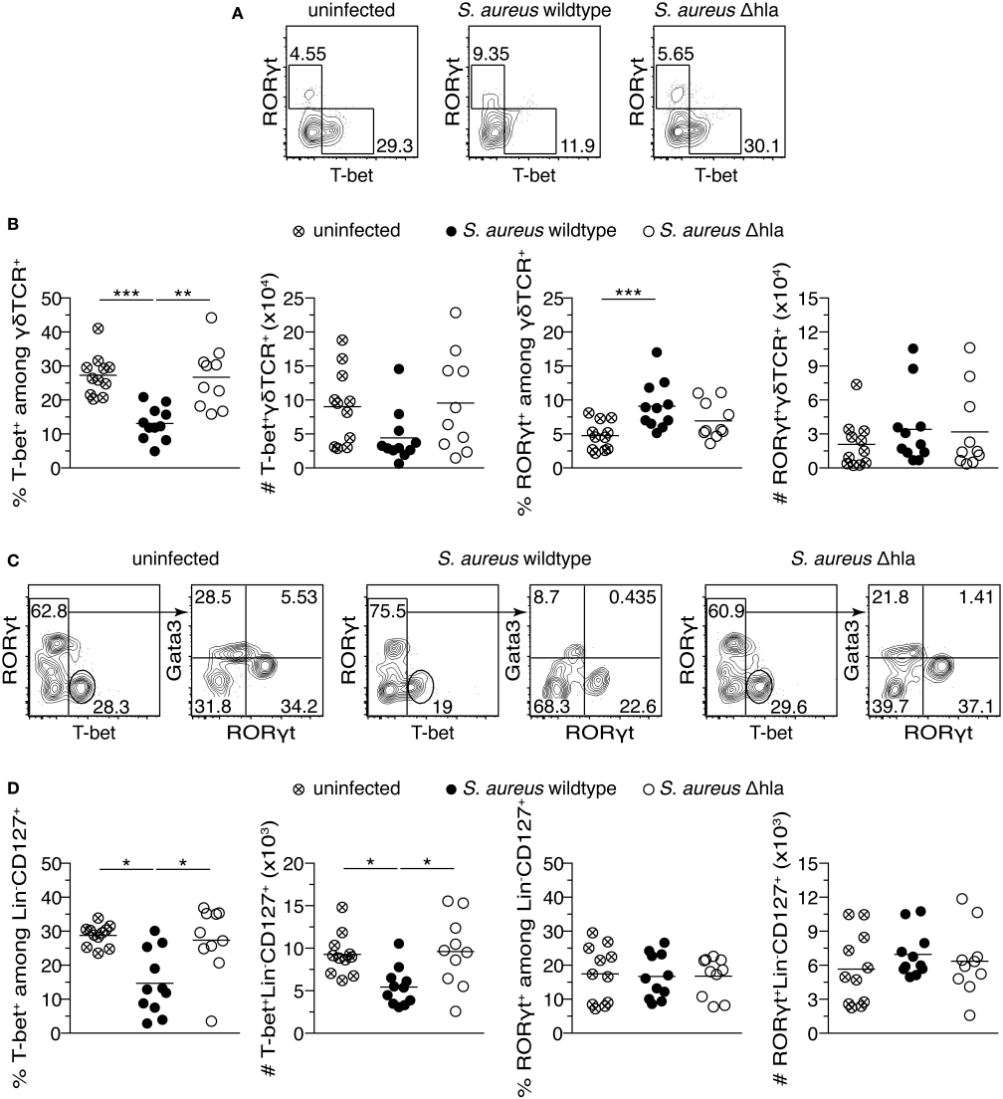
*S. aureus* alpha-toxin has a long-lasting impact on γδ T cells and ILC subsets. Female C57BL/6J mice were either left uninfected or infected i.v. with *S. aureus* wildtype or Δhla. On day 14 p.i., animals were sacrificed and organs harvested for flow cytometric analysis. Representative contour plots show T-bet and RORγt expression among splenic γδ T cells **(A)**. Frequencies (%) and absolute numbers (#) of T-bet^+^ and RORγt^+^ cells among γδ T cells in the spleen are depicted **(B)**. Representative contour plots show T-bet, Gata3, and RORγt expression among splenic ILCs **(C)**. Frequencies and absolute numbers of T-bet^+^ or RORγt^+^ ILCs in the spleen are depicted **(D)**. Data were pooled from two independent experiments and each dot represents one animal. For statistical analysis, Kruskal-Wallis test, followed by Dunn's multiple comparison test, was applied. *p < 0.05, **p < 0.01, ***p < 0.001.

After having demonstrated that alpha-toxin can modulate CD4^+^ T cells both *in vitro* and *in vivo*, we next aimed to assess if alpha-toxin is also able to modulate other immune cell types. To this end, we analyzed the abundance of γδ T cell and ILC subsets in mice infected with *S. aureus* wildtype vs. Δhla 14 days p.i. In spleens of mice infected with *S. aureus* wildtype, frequencies of RORγt^+^ γδ T cells were significantly increased, while T-bet^+^ γδ T cells were significantly decreased (Figures 5A,B and Figure S4B). Similar tendencies were observed for absolute cell numbers, yet not reaching statistical significance. In animals infected with *S. aureus* Δhla, T-bet^+^ γδ T cell frequencies and absolute numbers were similar to uninfected controls, while the RORγt^+^ subset was slightly, yet not significantly increased both in frequencies and absolute numbers as compared to uninfected controls. At the same time, we observed significantly decreased frequencies and absolute numbers of type 1 ILCs (T-bet^+^) in wildtype-infected animals as compared to *S. aureus* Δhla-infected animals and uninfected controls, while there was no impact of either infection on type 3 ILCs (RORγt^+^) (Figures 5C,D and Figure S4C). Together, we could show that alpha-toxin is also able to modulate other immune cell types including γδ T cells and ILCs, suggesting a more general effect of alpha-toxin on the modulation of type 1 and type 3 immune responses.

## Discussion

Alpha-toxin plays a major role during initial steps of infection by disrupting the epithelial barrier (38). In addition, it can affect signaling pathways in different cell types, hinting toward modulatory properties besides its cytolytic activity (19–21). While the innate immune response toward alpha-toxin has been studied in detail in different murine skin and wound infection models (6), the impact of alpha-toxin on the adaptive immune system, and more specifically on the differentiation of CD4^+^ T cells, is only incompletely understood. In the present study, we observed a superior resistance of Th17 cells toward alpha-toxin-induced cell death when compared to Th1 cells both *in vitro* and *in vivo*. Importantly, corresponding subsets of γδ T cells and ILCs were similarly affected *in vivo*, suggesting a more general effect of alpha-toxin on the modulation of type 1 and type 3 immune responses.

Besides its cytotoxic effect on different cell types, low concentrations of alpha-toxin have been reported previously to induce both IFNγ and IL-17A production in human PBMCs and CD4^+^ T cells (22–24). However, the CD4^+^ T cells were a mixture of memory and naïve CD4^+^ T cells in all studies, and IL-17A secretion upon stimulation with alpha-toxin could only be observed in Th17-polarized cells, suggesting that memory T cells were most probably the source of IFNγ and IL-17A production in alpha-toxin-stimulated CD4^+^ T cells. While we observed a differential impact of alpha-toxin on the survival of Th1 and Th17 cells, at the same time, the cytokine production was slightly enhanced in both subsets in the presence of alpha-toxin. As shown by Song et al., pore formation by alpha-toxin allows Ca^2+^ ions to enter the cell and induce downstream signaling (39), which could explain the abovementioned cytokine production upon stimulation of human T cells with sublytic concentrations of alpha-toxin (22, 23). In the same line, enhanced Ca^2+^ signaling might result in increased activation-induced cell death in Th1 cells, as these cells were reported to be more susceptible to Ca^2+^ signaling when compared to Th17 cells due to differential Fas ligand and c-FLIP expression as well as activity of p38 and ERK (40–42). Interestingly, we could mimic the differential effects of alpha-toxin on Th1- and Th17-polarized cells using Ionomycin, suggesting an increased susceptibility toward Ca^2+^ signaling in Th1 cells as mechanism underlying the differential susceptibility toward alpha-toxin-induced cell death. Yet, we could not observe a significant induction of caspases-3/7 in Th1-polarized cells cultured in presence of alpha-toxin, and Th1 cells were not rescued from alpha-toxin-induced cell death using a pan-caspase inhibitor, suggesting the involvement of caspase-independent mechanisms. To shed further light on the impact of alpha-toxin on Ca^2+^ signaling throughout the differentiation of Th1 and Th17 cells, one could utilize one of the described genetically encoded Ca^2+^ sensors (43–45).

Another possible explanation for the differential susceptibility of Th1 and Th17 cells toward alpha-toxin could be the varying expression levels of ADAM10, the cellular receptor for alpha-toxin (19). During differentiation, Th1 cells express slightly higher levels of ADAM10 as compared to Th17 cells. However, these subtle differences were not responsible for the increased susceptibility of Th1 cells toward alpha-toxin-induced cell death as blockade of alpha-toxin binding to ADAM10 by GI254023X did not significantly alter the survival of these cells. Noteworthy, ADAM10 is important for binding of alpha-toxin to the cell membrane at low concentrations, around 10 ng/ml, of the toxin, while it is not required at higher concentrations (19). It is tempting to speculate that a different membrane composition of Th1 and Th17 cells is accounting for the observed susceptibility toward alpha-toxin, yet this hypothesis needs to be tested in future experiments.

To assess the impact of alpha-toxin on CD4^+^ T cells *in vivo*, we have utilized a *S. aureus* bacteremia model, and in contrast to already published studies involving skin, wound or pulmonary infection models (46–48), bloodstream infection with wildtype *S. aureus* or an isogenic Δhla mutant strain caused similar disease severity as measured by body weight and survival, indicating comparable virulence of the two strains in this setting. Interestingly, we could recapitulate the *in vitro* finding by demonstrating a decreased frequency of T-bet^+^ CD4^+^ T cells when wildtype-infected were compared to Δhla-infected animals, while significantly increased frequencies of RORγt^+^ CD4^+^ T cells were observed upon infection with both strains. Interestingly, similar findings were made for γδ T cells, which are known to develop effector functions already in the fetal thymus and represent the main source of IL-17 under steady-state conditions (49–51). Previously, γδ T cells were reported to be the first and major source of IL-17 during *S. aureus* pneumonia and surgical site infection, and IL-17-producing γδ T cells were shown to be crucial for bacterial clearance (52, 53). Another subset of immune cells, namely ILCs, was also affected by *S. aureus* infection, and these effects were in part alpha-toxin-dependent. ILCs are mainly tissue-resident and, in contrast to T cells, lack antigen-specific receptors. They are responsive to various stimuli and are part of the immediate immune response. The three main subsets of ILCs—type 1, type 2, and type 3 ILCs—are innate counterparts to Th1, Th2, and Th17 cells, respectively. They can be identified by the same lineage transcription factors, and the factors promoting their activation are similar (54). Although ILCs act early during infection, we observed long-term effects of systemic *S. aureus* infection on type 1 ILCs. These cells were significantly decreased upon infection with *S. aureus*, and this effect was dependent on alpha-toxin. Interestingly, no long-term effects of systemic *S. aureus* infection on type 3 ILCs could be observed, while the corresponding subsets of CD4^+^ and γδ T cells were strongly affected. It is a possible scenario that type 3 ILCs were affected by the infection only during very early stages of the infection, and rapidly normalized in their frequencies until the later time point of analysis.

The molecular mechanism by which alpha-toxin affects T-bet^+^ cells among CD4^+^ T cells, γδ T cells and ILCs *in vivo* remains unknown. Also *in vivo*, alpha-toxin might have a direct negative effect on the survival of T-bet^+^ cells. However, we cannot exclude that alpha-toxin contributes indirectly, e.g., through the induction of inflammatory mediators by accessory cells. In this regard, it has been demonstrated that alpha-toxin can activate the NLRP3 inflammasome in monocytic cells (20), leading to production and secretion of IL-1β, which in turn can amplify Th17 differentiation as well as IL-17 production by γδ T cells (55). As a consequence, the resulting enhanced IL-17 production might have an effect on T-bet^+^ cells, as IL-17 can directly inhibit T-bet expression (56). Yet, in the present study we did not observe gross differences in systemic levels of inflammatory cytokines and chemokines, including IL-1β and IL-17, at day 14 p.i. when *S. aureus* wildtype and *S. aureus* Δhla infected animals were compared. However, we cannot exclude an impact of alpha-toxin on the induction of inflammatory mediators at earlier time points.

One question remains: Why does *S. aureus* produce alpha-toxin, as it is inducing an anti-bacterial type 3 immune response rather than inhibiting it? Firstly, alpha-toxin seems to be very important during initial steps of infection. In skin, wound or pulmonary infection models, immune cell infiltration and tissue damage were enhanced in presence of alpha-toxin, and its neutralization ameliorated disease severity (46–48, 57). The tuning of the adaptive immune response later in the infection process might be the trade-off that results from the differential responsiveness of the T cell subtypes to the formation of membrane pores. Secondly, the presence of Th1 cells has been shown to be critical for vaccine-mediated protection in different models (7–11, 58). Hence, the production of alpha-toxin might also be an immune evasion strategy by *S. aureus* to target the host's protective immunity by reduction of Th1 cells. From the host side, induction of IL-17 and subsequent recruitment of neutrophils is required for clearance of an *S. aureus* infection (59–61). On the other hand, patients suffering from *S. aureus* bloodstream infection and showing a high IL-17 immune response are more likely to die as compared to patients with a lower IL-17 response (62). However, death of the host is presumably not beneficial for the pathogen, and systemic infections with *S. aureus* are rare in comparison to local infections. Hence, alerting the host's immune system and induction of a mild anti-bacterial immune response via secretion of alpha-toxin might be a strategy to limit local infection and prevent systemic spread, promoting a long-lasting equilibrium between the pathogen and the host.

Taken together, we have identified alpha-toxin as a modulator of CD4^+^ T cells, γδ T cells, and ILCs, promoting type 3 while limiting type 1 immune responses. The knowledge gained from this study could be utilized in the future to develop novel therapeutic strategies against *S. aureus* or to selectively modulate immune responses toward type 3 immunity in different context.

## Data Availability Statement

All datasets generated for this study are included in the article/Supplementary Material.

## Ethics Statement

The animal study was reviewed and approved by Lower Saxony Committee on the Ethics of Animal Experiments (Lower Saxony State Office of Consumer Protection and Food Safety).

## Author Contributions

AB, OG, SF, SH, BB, EM, and JH conceptualized and designed the study. AB, OG, FK, PR, and MN performed the experiments. AB, OG, SF, SH, PR, ML, IS, BB, EM, FK, CF, and JH analyzed and interpreted the data. AB and JH wrote the manuscript. All authors contributed to the article and approved the submitted version.

## Conflict of Interest

The authors declare that the research was conducted in the absence of any commercial or financial relationships that could be construed as a potential conflict of interest.
