# Supplementary material for: Staphylococcus aureus Alpha-Toxin Limits Type 1 While Fostering Type 3 Immune Responses
Source: Front Immunol. 2020 Aug 7;11:1579. doi: 10.3389/fimmu.2020.01579 (PMC7427519; doi:10.3389/fimmu.2020.01579)
Supplement: Supplementary file 1 [file Data_Sheet_1.docx]

Supplementary Material

***Staphylococcus aureus* alpha-toxin limits type 1 while fostering type 3 immune responses**

**Agnes Bonifacius, Oliver Goldmann, Stefan Floess, Silva Holtfreter, Philippe A. Robert, Maria Nordengrün, Friederike Kruse, Matthias Lochner, Christine S. Falk, Ingo Schmitz, Barbara M. Bröker, Eva Medina, and Jochen Huehn**

## Supplementary Tables

| **Supplementary Table 1: Staining panels used in this study.**   \| **Panel** \| **Specificity** \| **Clone** \| **Fluorochrome** \| **Manufacturer** \| \| --- \| --- \| --- \| --- \| --- \| \| Sorting of  naïve CD4^+^ T cells \| hCD2 \| RPA-2.10 \| APC \| BioLegend \| \| CD4 \| RM4-5 \| PE/Cy7 \| BioLegend \| \| CD8a \| 53-6.7 \| APC \| BioLegend \| \| CD11c \| N418 \| APC \| BioLegend \| \| CD45R \| RA3-6B2 \| APC \| BioLegend \| \| CD62L \| MEL-14 \| BV605 \| BioLegend \| \| F4/80 \| BM8 \| APC \| BioLegend \| \| *in vitro*  Th1 differentiation \| LiveDead Fixable Blue \| \| \| Thermo Fisher Scientific \| \| CD4 \| RM4-5 \| PE/Cy7 \| BioLegend \| \| T-bet \| 4B10 \| PE \| eBioscience \| \| IFNγ \| XMG1.2 \| FITC \| BioLegend \| \| *in vitro*  Th17 differentiation \| LiveDead Fixable Blue \| \| \| Thermo Fisher Scientific \| \| CD4 \| RM4-5 \| PE/Cy7 \| BioLegend \| \| RORγt \| AFKJS-9 \| PE \| eBioscience \| \| IL-17A \| TC11-18H10.1 \| APC \| BioLegend \| \| Sorting of Tconv \| hCD2 \| RPA-2.10 \| APC \| BioLegend \| \| ADAM10 kinetic \| LiveDead Fixable Blue \| \| \| Thermo Fisher Scientific \| \| ADAM10 \| 139712 \| PE \| R&D Systems \| \| *in vivo*  CD4^+^ and γδ T cells \| LiveDead Fixable Blue \| \| \| Thermo Fisher Scientific \| \| CD3 \| 17A2 \| BV510 \| BioLegend \| \| CD4 \| RM4-5 \| BV605 \| BioLegend \| \| γδTCR \| eBioGL3 \| FITC \| eBioscience \| \| RORγt \| Q31-378 \| BV421 \| BD Biosciences \| \| T-bet \| 4B10 \| PE/Cy7 \| BioLegend \| \| IFNγ \| XMG1.2 \| BV785 \| BioLegend \| \| IL-17 \| eBio17B7 \| PE \| eBioscience \| \| *in vivo*  ILCs \| LiveDead Fixable Blue \| \| \| Thermo Fisher Scientific \| \| CD3 \| 17A2 \| APC \| BioLegend \| \| CD11c \| N418 \| APC \| BioLegend \| \| CD19 \| 6D5 \| APC \| BioLegend \| \| CD127 \| A7R34 \| PE \| BioLegend \| \| Gr1 \| RB6-8C5 \| APC \| BioLegend \| \| Ter119 \| TER-119 \| APC \| BioLegend \| \| RORγt \| Q31-378 \| BV421 \| BD Biosciences \| \| T-bet \| 4B10 \| PE/Cy7 \| BioLegend \| \| Gata3 \| TWAJ \| PerCP/eFluor710 \| eBioscience \| |
| --- | --- | --- | --- | --- | --- | --- | --- | --- | --- | --- | --- | --- | --- | --- | --- | --- | --- | --- | --- | --- | --- | --- | --- | --- | --- | --- | --- | --- | --- | --- | --- | --- | --- | --- | --- | --- | --- | --- | --- | --- | --- | --- | --- | --- | --- | --- | --- | --- | --- | --- | --- | --- | --- | --- | --- | --- | --- | --- | --- | --- | --- | --- | --- | --- | --- | --- | --- | --- | --- | --- | --- | --- | --- | --- | --- | --- | --- | --- | --- | --- | --- | --- | --- | --- | --- | --- | --- | --- | --- | --- | --- | --- | --- | --- | --- | --- | --- | --- | --- | --- | --- | --- | --- | --- | --- | --- | --- | --- | --- | --- | --- | --- | --- | --- | --- | --- | --- | --- | --- | --- | --- | --- | --- | --- | --- | --- | --- | --- | --- | --- | --- | --- | --- | --- | --- | --- | --- | --- | --- | --- | --- | --- | --- | --- | --- | --- | --- | --- | --- | --- | --- | --- | --- | --- | --- | --- |

## Supplementary Figures

| **Figure S1** |
| --- |
| 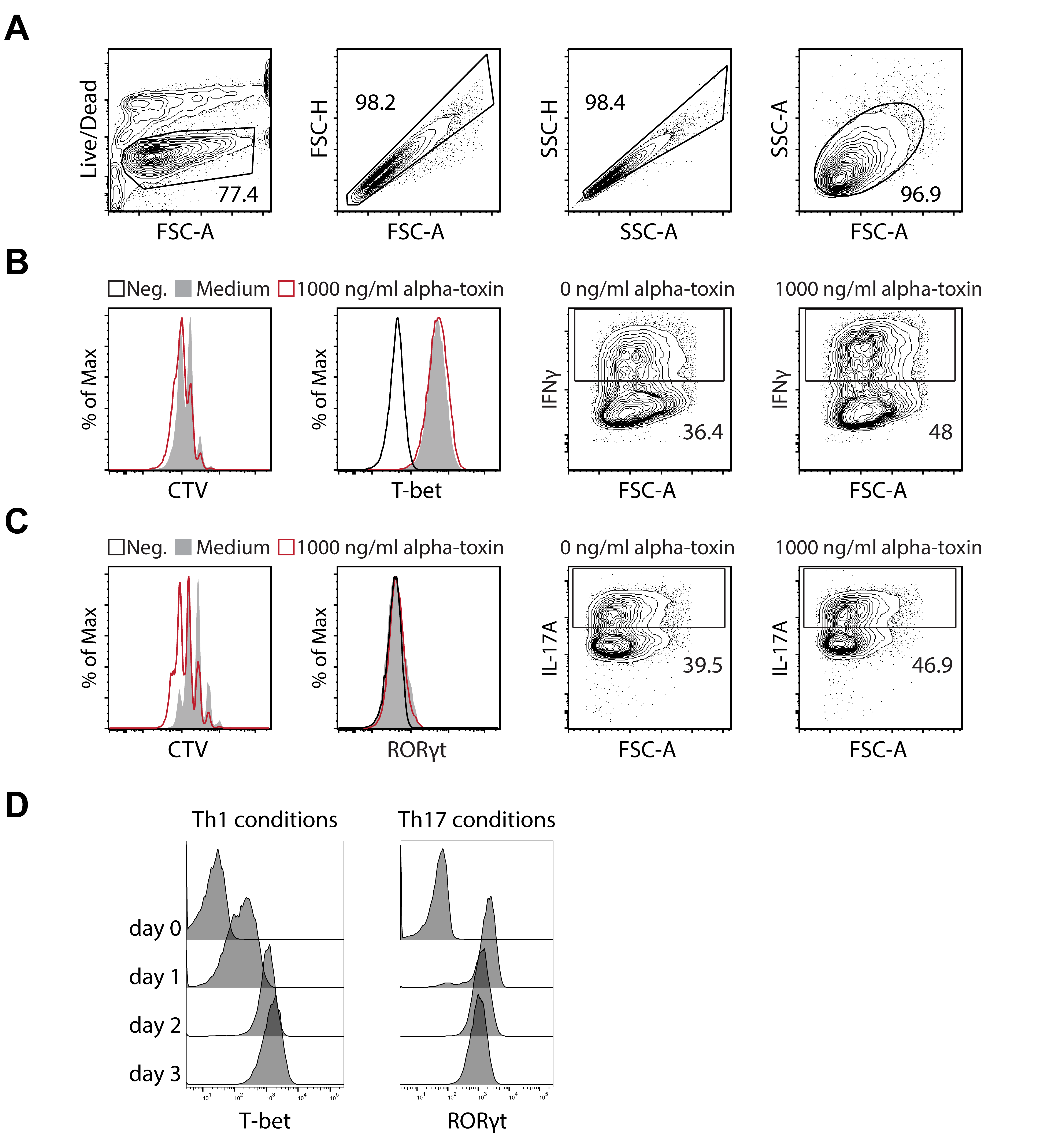 |
| **Figure S1: Gating strategy and representative staining of Th1 and Th17 cells differentiated in presence or absence of alpha-toxin.** Murine naïve CD4^+^ T cells were differentiated into Th1 or Th17 cells in presence of indicated concentrations of alpha-toxin. The cells were analyzed by flow cytometry at day 4 of the culture if not stated otherwise. (**A**) The gating strategy is depicted by exemplary contour plots. (**B**) Representative histograms and contour plots of Th1-polarized cells show the proliferation marker CellTrace™ Violet (CTV), as well as T‑bet and IFNγ expression. (**C**) Representative histograms and contour plots of Th17-polarized cells show the proliferation marker CTV, as well as RORγt, and IL‑17A expression. (**D**) Cells cultured in 24 well plates under Th1 (left) or Th17 (right) conditions were harvested for flow cytometric analysis at indicated time points. Data are representative of three independent experiments. |

**Figure S2**


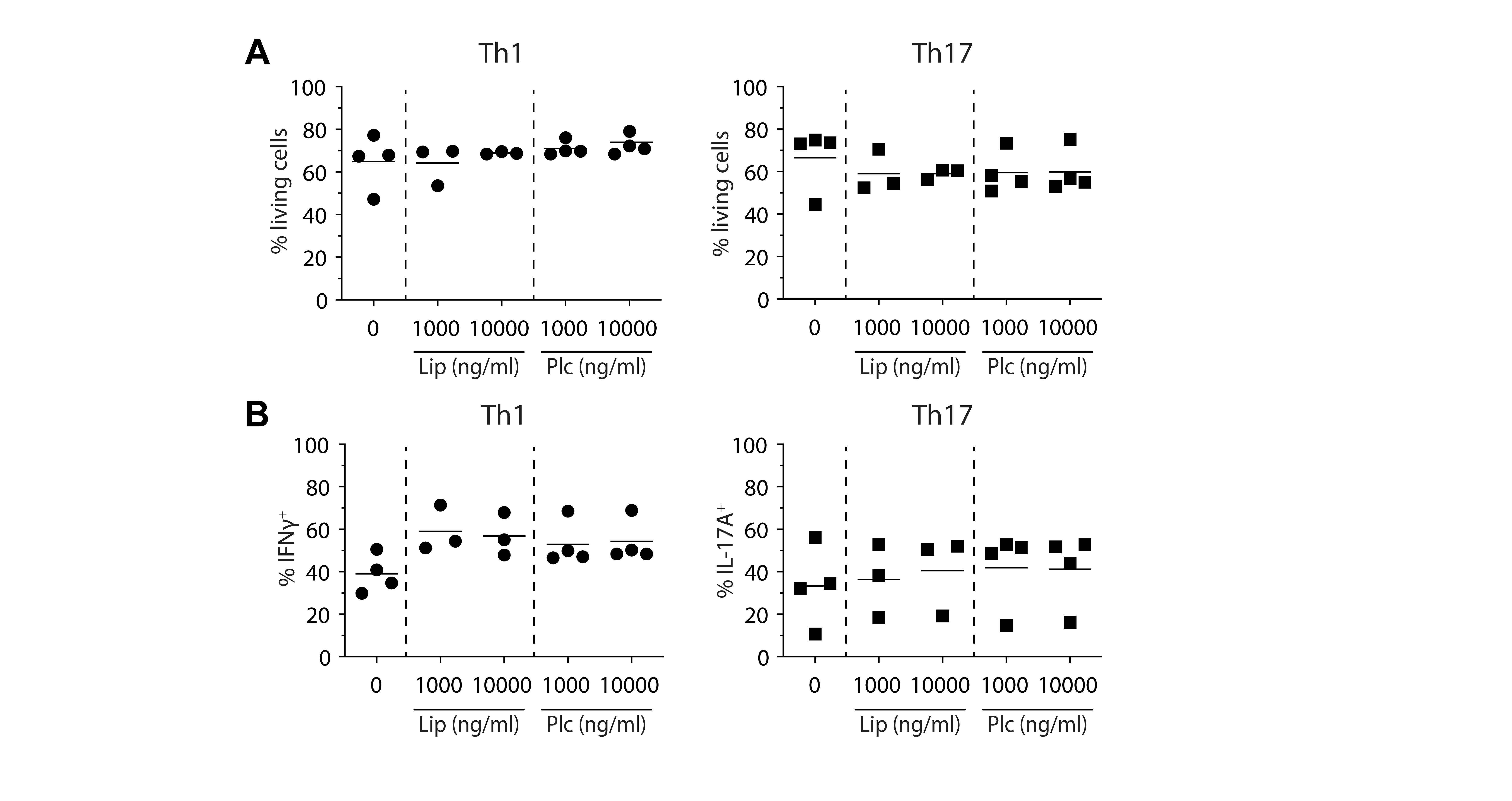


**Figure S2: Impact of Plc and Lip on Th1 and Th17 cell differentiation.** Murine naïve CD4^+^ T cells were differentiated into Th1 or Th17 cells in presence of indicated concentrations of Plc or Lip. On day 4 of the culture, the cells were analyzed by flow cytometry. (**A**) Summarizing graphs show the frequency of living cells. (**B**) Summarizing graphs show the frequency of IFNγ- or IL-17A-producing cells, respectively. Data were pooled from 3-4 independent experiments with technical triplicates.

**Figure S3**

**Figure S3: Impact of alpha-toxin on survival of already differentiated Th1 and Th17 cells.** Murine naïve CD4^+^ T cells were differentiated into Th1 or Th17 cells. On day 3, cells were removed from the stimulus and rested in presence of IL‑2 or IL‑23, respectively. On day 5, cells were either kept in culture without restimulation or restimulated with anti‑CD3 and anti‑CD28. Part of the cells received alpha-toxin, and cell survival was analyzed via flow cytometry after 24 or 48 hours. Data were pooled from two independent experiments with technical triplicates.

| **Figure S4** |
| --- |
| 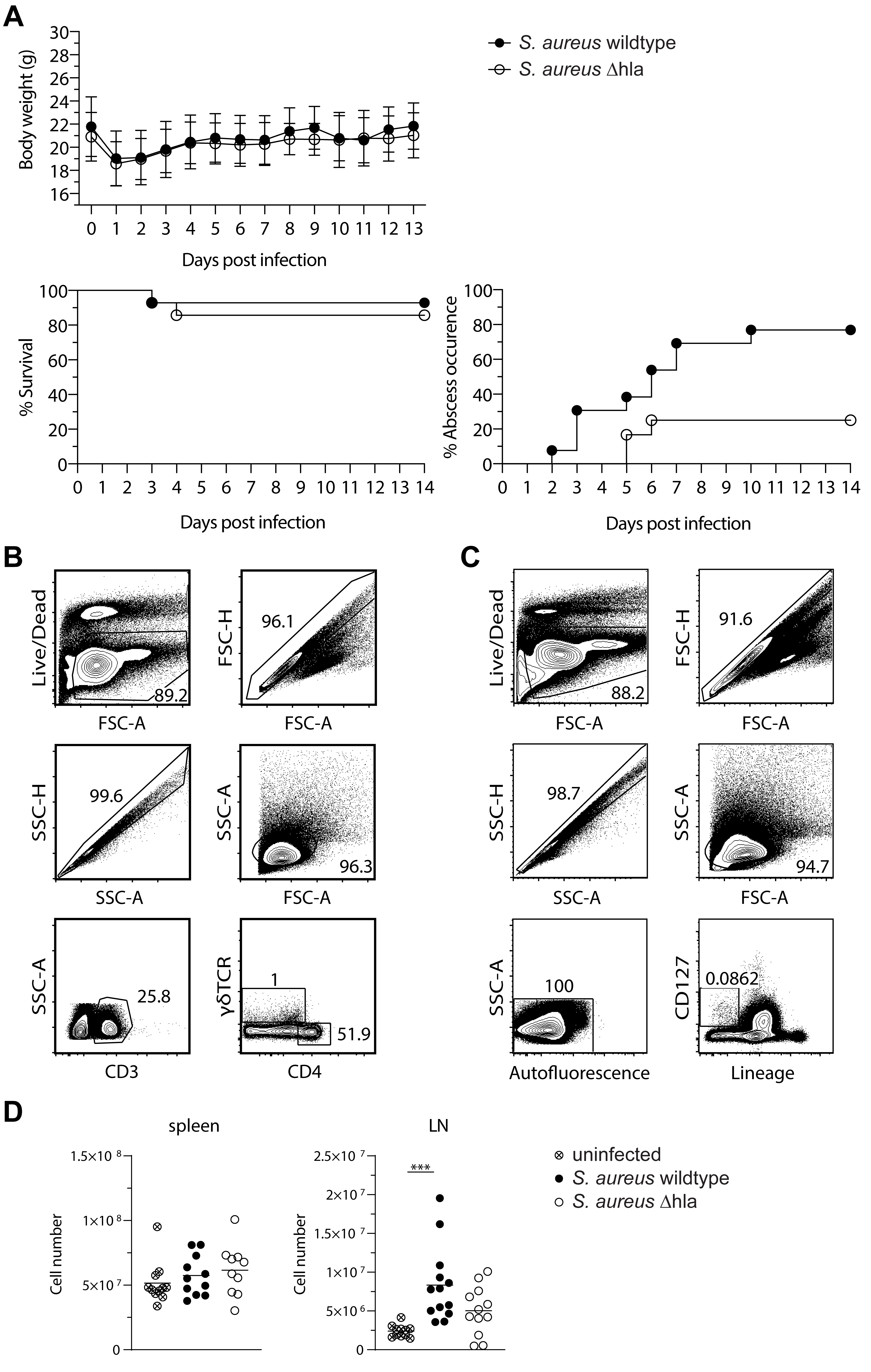 |
| **Figure S4: Disease progression during systemic *S. aureus* infection and gating strategy for flow cytometric analysis.** (Legend on next page) |

**Figure S4: Disease progression during systemic *S. aureus* infection and gating strategy for flow cytometric analysis.** Female C57BL/6J mice were either left uninfected or infected i.v. with *S. aureus* wildtype or Δhla. On day 14 p.i., animals were sacrificed and organs harvested for flow cytometric analysis. (**A**) Graphs show body weight (mean±SD), survival, and superficial tissue lesion (abscess) development during the course of infection. Data were pooled from two independent experiments and fourteen animals were included per group. For body weight and abscess development graphs, only data from surviving animals were included. (**B**) Exemplary contour plots from a spleen sample showing the gating strategy for the CD4^+^ T cell and the γδ T cell compartment. (**C**) Exemplary contour plots from a spleen sample showing the gating strategy for the ILCs. (**D**) Total cell counts of spleens and LNs from uninfected animals or animals infected with *S. aureus* wildtype or Δhla were determined. Data were pooled from two independent experiments and each dot represents one animal. For statistical analysis, Kruskal-Wallis test, followed by Dunn’s multiple comparison test, was applied. *** p < 0.001.

**Figure S5**

**Figure S5: Cytokine and chemokine levels in serum of uninfected or *S. aureus* infected mice.** Female C57BL/6J mice were either left uninfected or infected i.v. with *S. aureus* wildtype or Δhla. On day 14 p.i., animals were sacrificed and blood was collected for measurement of cytokines and chemokines in serum. Data were pooled from two independent experiments and each dot represents one animal. For statistical analysis, Kruskal-Wallis test, followed by Dunn’s multiple comparison test, was applied. * p < 0.05, ** p < 0.01.

| **Figure S6** |
| --- |
|  |
| **Figure S6: *S. aureus* alpha-toxin induces IL-17A-producing CD4^+^ helper T cells.** Female C57BL/6J mice were either left uninfected or infected i.v. with *S. aureus* wildtype or Δhla. On day 14 p.i., animals were sacrificed and organs harvested for flow cytometric analysis. (**A**) Representative contour plots show IFNγ and IL‑17A expression among CD4^+^ T cells. (**B**) Frequencies of IFNγ^+^ cells among CD4^+^ T cells in spleen and LNs are depicted. (**C**) Frequencies of IL‑17A^+^ cells among CD4^+^ T cells in spleen and LNs are depicted. (**B+C**) Data were pooled from two independent experiments and each dot represents one animal. For statistical analysis, Kruskal-Wallis test, followed by Dunn’s multiple comparison test, was applied. * p < 0.05, *** p < 0.001. |
